# Predicting childhood obesity using electronic health records and publicly available data

**DOI:** 10.1371/journal.pone.0215571

**Published:** 2019-04-22

**Authors:** Robert Hammond, Rodoniki Athanasiadou, Silvia Curado, Yindalon Aphinyanaphongs, Courtney Abrams, Mary Jo Messito, Rachel Gross, Michelle Katzow, Melanie Jay, Narges Razavian, Brian Elbel

**Affiliations:** 1 NYU Langone Comprehensive Program on Obesity, NYU School of Medicine, New York, New York, United States of America; 2 Department of Cell Biology, NYU School of Medicine, New York, New York, United States of America; 3 Department of Population Health, NYU School of Medicine, New York, New York, United States of America; 4 Department of Pediatrics, NYU School of Medicine, Bellevue Hospital Center, New York, New York, United States of America; 5 Department of Medicine, NYU School of Medicine, New York, New York, United States of America; 6 Department of Radiology, NYU School of Medicine, New York, New York, United States of America; 7 NYU Wagner Graduate School of Public Service, New York, New York, United States of America; Ben-Gurion University of the Negev, ISRAEL

## Abstract

**Background:**

Because of the strong link between childhood obesity and adulthood obesity comorbidities, and the difficulty in decreasing body mass index (BMI) later in life, effective strategies are needed to address this condition in early childhood. The ability to predict obesity before age five could be a useful tool, allowing prevention strategies to focus on high risk children. The few existing prediction models for obesity in childhood have primarily employed data from longitudinal cohort studies, relying on difficult to collect data that are not readily available to all practitioners. Instead, we utilized real-world unaugmented electronic health record (EHR) data from the first two years of life to predict obesity status at age five, an approach not yet taken in pediatric obesity research.

**Methods and findings:**

We trained a variety of machine learning algorithms to perform both binary classification and regression. Following previous studies demonstrating different obesity determinants for boys and girls, we similarly developed separate models for both groups. In each of the separate models for boys and girls we found that weight for length z-score, BMI between 19 and 24 months, and the last BMI measure recorded before age two were the most important features for prediction. The best performing models were able to predict obesity with an Area Under the Receiver Operator Characteristic Curve (AUC) of 81.7% for girls and 76.1% for boys.

**Conclusions:**

We were able to predict obesity at age five using EHR data with an AUC comparable to cohort-based studies, reducing the need for investment in additional data collection. Our results suggest that machine learning approaches for predicting future childhood obesity using EHR data could improve the ability of clinicians and researchers to drive future policy, intervention design, and the decision-making process in a clinical setting.

## Introduction

Childhood obesity has been increasing since the 1970s [1]. As of 2016, 18.5% of US children and adolescents aged 2–19 had obesity, with a significantly higher prevalence among boys than girls [2]. Although there has been recent cause to suspect obesity rates for adults and children might be leveling off [3, 4], more recent data question this conclusion [5]: data from 2015–2016 showed increases in obesity rates across children of all ages, including a large increase among children at the youngest ages, 2–5 years old [2]. Growth trajectory simulation models suggest that 57% of children today will have obesity at age 35 [6]. This upward trend is concerning as childhood obesity can lead to diabetes, hypertension, and other conditions in adulthood [7–9]. Because of the strong link between childhood obesity and adult comorbidities, and the difficulty in decreasing BMI later in life, effective strategies are needed to address the condition early in life. In fact, a growing number of early obesity prevention interventions are being developed to decrease obesity-promoting feeding and lifestyle practices beginning in pregnancy and infancy. Some are beginning to demonstrate promising impacts on both promoting healthy habits and decreasing early childhood obesity; however, they currently focus on universal interventions [10–18]. If we were instead able to predict the risk level of a child developing obesity, we would then be able to better target intervention resources through the measurement of the effect of an intervention relative to a child’s risk of developing obesity.

**Fig 1:**
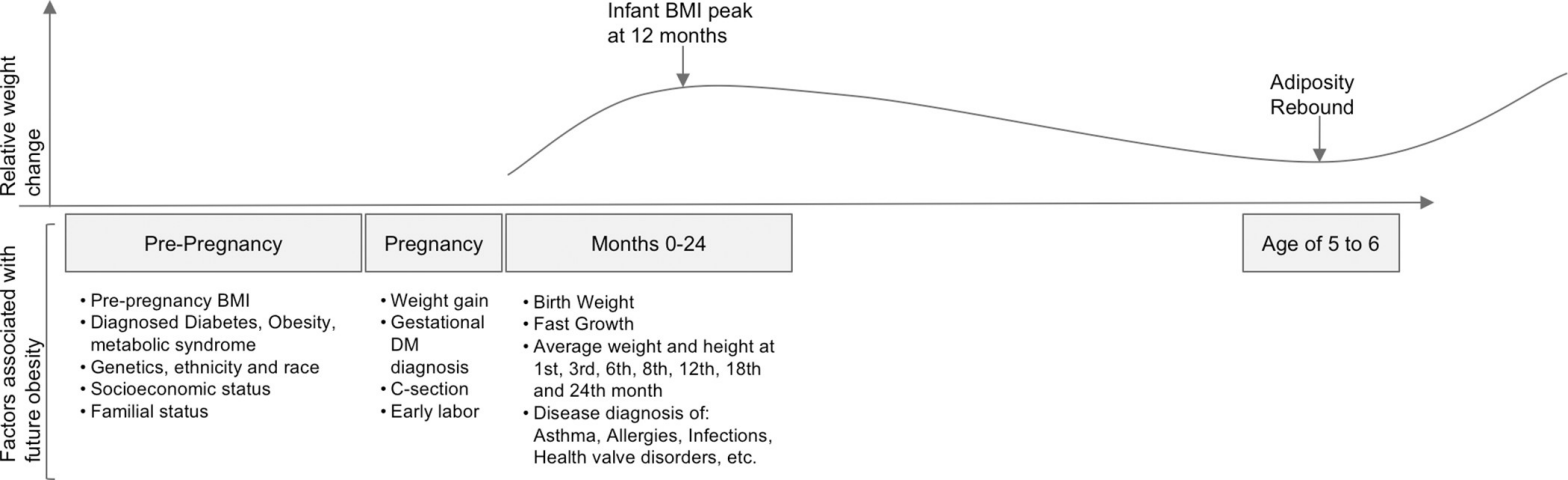
Factors at the prenatal and infancy periods associated with early childhood obesity by age five. Adapted from González-Muniesa et al. [21].

Two critical periods in the development of obesity include the prenatal and infancy period, and early childhood (Fig 1). The first 1,000 days [19, 20], from conception until the end of the second year of life, mark the first critical period in the development of obesity. The second period starts at age five, where the adiposity rebound marks a BMI minimum and a shift into childhood growth.

Obesity during the early childhood critical period significantly increases the risk of obesity later in life [22, 23]. The ability to predict obesity before age five could be a useful tool, allowing prevention strategies to focus on children with a high risk of developing obesity. Primary care represents a promising platform for early childhood obesity prevention given the high frequency of visits during pregnancy and infancy, which provides access to infants and pregnant women. Additionally, a number of prenatal and infancy conditions are known risk factors for obesity at age five (Fig 1) [21].

Risk factors previously associated with childhood obesity range from 1) individual and parental biological factors, such as the infant’s birth weight [24–27], microbiome composition [28], maternal factors (including health diagnoses and weight gain), to 2) other family influences, such as race/ethnicity [29] and income [30]), and 3) neighborhood-level factors [31] (e.g., food availability, crime, and built environment). However, because of the complexity of the disease, this list likely still misses unknown key factors as well as the overall interdependence between the already identified determinants, making it challenging to predict with precision a child’s risk for developing obesity.

There are a few existing prediction models for obesity in infants, children, and adolescents that have primarily used data from prospective longitudinal cohort studies, and tend to employ traditional statistical methods, not machine learning approaches [32–37]. These studies demonstrated that it is possible to predict obesity during critical developmental periods, and offered quantitative insights on how different covariates correlated with key outcomes. However, these models are not generalizable to a broad clinical setting, given the high costs of data collection, and the fragility of those models in the cases of missing variables or small inaccuracies. This limitation significantly impacts the generalizability of predictions offered by such studies.

As of 2015, EHR systems were implemented in 84% of all US hospitals and approximately 87% of physicians’ offices [38–40]. Their widespread adoption also means that medical histories for each patient can be readily available for quantitative analysis at limited additional cost. In medicine, machine learning approaches have already seen successes, for example, in diagnostic medical imaging [41, 42], drug target discovery [43], early prediction of sepsis [44], type 2 diabetes [45], multiple families of diseases [46–50], and patient selection for clinical trials [51, 52]. There have been some machine learning approaches to predict later childhood obesity; however, research in this field is limited and there still exist a significant number of open issues. The most related research to our project is Dugan, et al. [53] which is explained in further detail below; other existing studies include some that proposed algorithms but without reporting results, leaving no point of comparison for future work [54–56]; another used data for children between ages 9 and 11, reducing its clinical utility to stop the development of obesity [57]; one that only utilizes 12 children, which gives insight into a small set of children but cannot be generalized to a broader population [58]; and two studies that compare a wide range of commonly used algorithms in machine learning, but report a static set of metrics, making it hard to compare performance across a set of metrics [53, 59]. The work of Dugan, et al. [53], however, demonstrated that it is possible to train machine learning models for obesity prediction using data from a custom clinical decision support system that incorporates both precise measurements and questionnaire data in a safety net hospital system in Indiana. Their work found race, the development of overweight between the ages of one and two, and accelerated weight gain to be important factors for prediction [53].

Similar to Dugan, et al. [53], our study used existing EHR data from the first critical period (pre-pregnancy through age two) from a safety net health system (ours in New York City) to predict future childhood obesity using machine learning. The substantive differences are that 1) we aimed to predict obesity at age five where adiposity is at a minimum during development, compared to obesity occurring at some point between the ages of two and ten, 2) we focused on reporting results across a sliding scale for the risk of developing obesity at age five, as opposed to all children who will become obese, as knowing the risk earlier may help to guide intervention studies, and 3) we used standard EHR data combined with census data, which requires no additional work from the clinician during a visit, rather than EHR data with supplementary, site-specific questionnaire data. Although EHR datasets are often noisy and incomplete due to numerous issues such as data entry errors and selective form fills, our model’s ability to make predictions using EHR data may allow for the approach to be more widely implemented, as it avoids the limitations of expensive cohort studies. Because machine learning models can be more effective than traditional statistical methods in handling missing, noisy, and asymmetric data (a common limitation of EHR data) we argue that our study can become more widely applicable in a clinical setting for guiding intervention efforts, compared to those that require the use of highly accurate and symmetrically collected cohort data, which is often not possible in a clinical setting due to resource limitations.

## Methods

We conducted a retrospective cohort study using EHR data from patients in a safety net health system that serves a racially and ethnically diverse urban community in New York City: Family Health Centers at NYU Langone (formerly, Lutheran Family Health Centers)—one of the largest Federally Qualified Health Centers in the U.S.—which is composed of 8 primary care and specialty locations and over 40 school-based clinics in Brooklyn, New York. The EHR data employed by this study spanned from January 1, 2008 to August 31, 2016 and contained the records of 52,945 children of various ages, and 36,244 of their respective mothers for visits ranging from well-child visits to inpatient and outpatient services. Because not all mothers had given birth or received care in the study health system, there was not always a one-to-one match between mothers and their children. Additionally, some mothers had given birth to more than one child during the data collection period, also contributed to a lower number of mothers represented in the data set than children. The work was approved by the New York University School of Medicine’s Institutional Review Board and we were granted a waiver of informed consent as well as a waiver of authorization to use private health information for research.

The first set of criteria for a child to be considered in our study was to have at least one BMI measurement between the ages of 4.5 and 5.5 years (11,494 children) and be in the range of 10–40 kg/m^2^ (11,484 children), values outside of the CDC reference table minimums and maximums, to ensure there were no erroneous data points [60]. The second was that each child had to have at least one visit in the first two years of life (5,746 children). The third was to have the mother’s data available (3,451 children). When all three criteria are combined our study cohort shrunk to 3,449 children (1,751 boys, and 1,698 girls). Table 1 summarizes the effect of these three inclusion criteria used for this study on the full dataset. We included all children who passed our selection criteria for both modeling and prediction. As such, our selected cohort is not intended to be a random population sample.

**Table 1:** Number of children included at each selection criteria.

Selection Criteria (in order)	N Boys	N Girls	N
1) Full data set	26,507	26,438	52,945
2a) BMI reading between 4.5 and 5.5 years	5,775	5,719	11,494
2b) BMI reading is valid	5,770	5,714	11,484
3) At least one data point prior to 2 years of age	2,860	2,886	5,746
4) Maternal data available	1,751	1,700	3,451
**Study Cohort Final**	**1,751**	**1,698**	**3,449**

### Feature engineering

The EHR data used in this analysis—from both children and respective mothers—included the following features for each of their encounters or visit to a healthcare facility, for any purpose, in the study health system: demographic information (ethnicity, race, country of origin, nationality, and languages spoken), home address (allowing us to determine zip code and census tract), vital signs, medications, all laboratory test orders and results, diagnosis codes, and all medical procedures administered.

For maternal data we used vital signs, diagnosis codes, procedure, and laboratory results during six separate time periods: pre-pregnancy (prior to 40 weeks before birth), first trimester (0–14 weeks before birth), second trimester (14–27 weeks before birth), third trimester (27–40 weeks before birth), post-pregnancy, and during any other pregnancy. Taking these six time periods into account separately allowed us to understand the potential relationships between maternal health before, during, or after pregnancy and the child’s growth. For all other EHR data, such as delivery age or ethnicity, we only created one feature for each possibility as they do not change over time.

For the children’s data, we created features that group vital signs into averages over 11 time periods: at birth, 0–1 months, 1–3 months, 3–5 months, 5–7 months, 7–10 months, 10–13 months, 13–16 months, 16–19 months, 19–24 months, and latest measurements available (before 24 months), to capture the timeframes surrounding the standard well-child visits during the first two years of life [61]. Additionally, we calculated the change between each of these time periods as well as the change from birth to age two for all vital signs. For all other EHR data—diagnosis codes, demographic data, labs, or medications—we only created a single feature for each of the individual variables in the two-year time frame. For any data point that was not available, we filled in the corresponding matrix value with a zero.

Further, using the Clinical Classification Software categories, we collapsed all of the International Classification of Diseases 9^th^ Revision diagnosis codes into 283 standard disease groupings to account for multiple related diagnosis codes. We then created binary encodings for all of the disease groups to indicate the presence of a diagnosis during each of the aforementioned time periods for mothers and for children at any point during the first two years of childhood. For lab results and vitals, we considered the average value for the maternal and childhood time periods. For features where we only considered whether or not they exist, i.e., medications, procedures, and demographic information, we created binary variables to indicate their presence.

Given the likely role of neighborhoods in the development of obesity [31], we also examined 17 continuous features at the census tract level derived from 2015 American Community Survey 5-year Estimates by geocoding each child’s address closest to birth and age two, using the NYCgbat Geosupport Desktop Edition [62]. These tract-level features included: percentage of population with a disability, education level, percentage of households participating in Supplemental Nutritional Assistance Program (SNAP, or food stamps), unemployment rate, and median household income. In addition, we created a binary variable for each of the 652 unique residential zip codes in the data to determine if there were any zip code-level influences not picked up by the census tract characteristics.

A total of 19,290 variables were created from all of the mentioned feature categories (e.g., diagnosis codes, labs, and ethnicity) for use in this analysis from all of the EHR data available combined with the census data. In Table 2, we show the number of features by category. The size of the feature space was a result of the sheer number of possible diagnosis codes, lab tests, and medications available. However, this did not necessarily translate to a positive impact on modeling because our feature space shrunk to 12% of the original 19,290 features when we look at variables that contain any information, and to 8% when we consider features with enough information to be useful (minimum of five children with information for a given variable). Many of these features are rare to begin with, such as most diagnosis codes or medications, however, for other features, there was likely genuinely missing information in our records. In addition, in the Jupyter Notebook in S1 File all of the generated features are included with number of occurrences and descriptive statistics for the overall data and valid cohort (combined and separated by gender).

**Table 2:** Number of features by category.

Feature Category	Number of Features	Number of Features with at Least 1 Occurrence	Number of Features with at Least 5 Occurrences
Diagnosis	566	160	107
Lab	549	73	57
Medication	2,968	78	14
Gender	2	2	2
Ethnicity	2	2	2
Race	11	9	8
Vital	475	255	255
Number of visits	1	1	1
Zip code	652	207	86
Census	34	34	34
Maternal diagnosis	3,962	473	257
Newborn diagnosis	566	52	22
Maternal ethnicity	4	3	3
Primary insurance	419	67	29
Secondary insurance	120	16	3
Maternal race	7	7	5
Maternal language	30	7	5
Maternal nationality	126	61	24
Maternal marriage status	7	5	5
Maternal birthplace	142	56	23
Maternal delivery age	1	1	1
Maternal lab history	5,700	573	477
Maternal procedure history	2,946	169	89
**Total**	**19,290**	**2,311**	**1,509**

### Outcome definition

To predict obesity, we first calculated the BMI percentile by age, in months, and gender per the Center for Disease Control and Prevention (CDC) guidelines, for each BMI reading between the ages of 4.5 and 5.5 years [60]. If more than one record was available, we computed the median age, BMI, and BMI percentile as the final reading. We then determined obesity status by creating a binary variable to indicate whether or not a child is obese as defined by the CDC: BMI percentile being greater than or equal to the 95^th^ percentile, according to the standard percentiles defined in [60].

### Analysis methods

We used both regression and classification techniques for predicting childhood obesity. In the classification task, we used class probabilities to predict the binary outcome of obesity status: obese/not obese. In the regression task we normalized the median BMI value, as is standard practice for continuous variables. Using the predicted normalized BMI, we classified children as having obesity if they had a predicted value greater than the threshold for obesity.

For predicting our dichotomous measures of obese/not obese we used logistic regression with L1 loss, a random forest classifier, and gradient boosting classifier. For predicting our continuous BMI values we employed LASSO regression, random forest regression, and gradient boosting regression. These algorithms were the implemented versions in Python’s Scikit-learn package (version 0.19.1) [63]. LASSO regression and logistic regression were used as a baseline for machine learning performance. Random forest and gradient boosting were chosen because of their reported high performance across many tasks, especially those with a large feature space such as our own. As is standard practice, we normalized all of our continuous features before training each algorithm by subtracting the mean from each value and dividing by the standard deviation, respective to the values column mean and standard deviation.

To assess the performance of each of our models we randomly selected 20% of our data (350 boys and 339 girls) to be held out as a test set for all analyses for maintaining a consistent comparison of performance. Using the remaining data, we used bootstrap cross validation to validate our models by randomly sampling 90% of the data in each iteration without replacement, then performing a 70%/30% split for training and validation, and utilized our test data to assess final performance. Bootstrapping allowed us to compute the average AUC, along with a 95% confidence interval and represented a more real-world scenario for model implementation as opposed to a k-fold cross validation. For the comparison of classification and regression models 20 bootstraps were used. Final results on the best performing set of models were further refined by running 100 bootstraps.

For each of the regression and classification algorithms, we performed a series of feature selection techniques to further refine our methods and to test the effects that certain categories of features had on performance. In total, there were 13 variations of the data for each of the boys’ and girls’ cohorts that were used to train a model for each of the three regression and classification algorithms, making a total of 156 analyses. To create the 13 variations, we combined three category-based feature sets and three feature selection techniques. The three feature sets were: the full feature set (including variables with no information), only EHR features (which exclude census and zip code features), and non-weight or BMI features; the three feature selection methods consisted of no feature selection, features with at least five non-zero entries, and 10 bootstrap LASSO feature selection. In the LASSO feature selection, we selected all features whose average feature weight was non-zero in a 10 bootstrap LASSO regression process. We then created nine feature sets by considering all possible combinations of feature selection and feature category-based subset methods. The remaining four models used single features, and acted as a baseline of performance, given their importance to childhood obesity: the average weight for length (WFL) z-score between 19 and 24 months, the latest WFL available before 24 months, the average BMI between 19 and 24 months, and the latest BMI reading available before 24 months. Although WFL is more clinically meaningful for assessing childhood obesity, it has been suggested that BMI-z is more closely associated with later childhood obesity than WFL from a prediction standpoint [64], and thus we have incorporated both.

## Results

The first column of Table 3 shows the demographic breakdown of our EHR population prior to applying our inclusion criteria. These results are comparable to our modeling cohorts with the exceptions of the “No Data Available” categories. Using all 3,449 children (1,751 boys and 1,698 girls) in the study cohort (Table 1) we assessed each variable’s association with the binary obesity outcome between the ages of 4.5 and 5.5. We compared these associations with obesity to the reference group (defined in each feature category section) and show a subset of those variables in Table 3. Overall, 18.6% of our cohort was obese at age five, which is less than the NYC estimate of children attending public schools in grades Kindergarten through eighth grade of 21% [31]. Only a single diagnoses category had a significant association (p<0.001) with obesity at age five: maternal diabetes mellitus, with no infant diagnoses determined to have had a significant association with obesity.

**Table 3:** Individual feature associations with obesity between ages 4.5 and 5.5.

Variable	% of EHR Population	Girls			Boys		
		% of Cohort	Odds Ratio (95% CI)	p-value for OR	% of Cohort	Odds Ratio (95% CI)	p-value for OR
**Total Number**	52,945	1,698	-	-	1751	-	-
**Ethnicity**	** **	** **	** **	** **	** **	** **	** **
Not Hispanic/Latina	24%	17%	0.587 (0.395, 0.874)	0.009	21%	0.714 (0.529, 0.963)	0.027
Hispanic/Latino	49%	82%	1.546 (1.053, 2.269)	0.026	79%	1.399 (1.039, 1.884)	0.027
Other/Not Reported	27%	0%	-	-	0%	-	-
**Race**	** **	** **	** **	** **	** **	** **	** **
Caucasian/White	15%	5%	1.151 (0.65, 2.038)	0.630	4%	0.827 (0.482, 1.421)	0.492
African Amer/Black	13%	5%	1.913 (1.119, 3.27)	0.018	5%	0.907 (0.526, 1.565)	0.725
Asian	10%	9%	0.204 (0.089, 0.466)	p<0.001	10%	0.623 (0.419, 0.925)	0.019
Multiracial	42%	77%	1.085 (0.786, 1.497)	0.622	68%	1.283 (0.979, 1.681)	0.071
Other	14%	3%	1.828 (0.965, 3.462)	0.064		1.321 (0.748, 2.332)	0.337
Unknown/No Response	6%	0%	-	-	0%	-	-
**Maternal Marriage Status**	** **	** **	** **	** **	** **	** **	** **
Married	7%	36%	0.702 (0.526, 0.938)	0.017	36%	0.875 (0.689, 1.111)	0.272
Divorced	0%	0%	1.885 (0.378, 9.389)	0.439	1%	2.546 (0.804, 8.067)	0.112
Partnered	4%	32%	1.135 (0.856, 1.504)	0.379	30%	1.067 (0.836, 1.363)	0.601
Single	6%	31%	1.15 (0.867, 1.524)	0.332	33%	1.057 (0.832, 1.343)	0.651
Other/Unknown/No Response	0%	1%	5.725 (1.645, 19.92)	0.006		0.587 (0.131, 2.635)	0.487
No Data Available	83%	0%	-	-	0%	-	-
**Maternal Birthplace**	** **	** **	** **	** **	** **	** **	** **
United States	3%	12%	1.436 (0.992, 2.081)	0.055	13%	1.041 (0.749, 1.447)	0.810
China	2%	9%	0.25 (0.115, 0.539)	p<0.001	11%	0.64 (0.431, 0.951)	0.027
Dominican Republic	1%	3%	1.681 (0.871, 3.243)	0.122	4%	2.369 (1.441, 3.896)	p<0.001
Ecuador	1%	5%	2.443 (1.475, 4.048)	p<0.001	5%	1.011 (0.591, 1.729)	0.968
Mexico	7%	53%	0.788 (0.604, 1.028)	0.079	50%	1.078 (0.86, 1.351)	0.515
El Salvador	0%	3%	1.425 (0.703, 2.887)	0.326	3%	1.011 (0.496, 2.06)	0.977
Guatemala	1%	5%	0.589 (0.292, 1.187)	0.139	5%	0.695 (0.401, 1.203)	0.193
Other	2%	10%	1.418 (0.94, 2.139)	0.096		0.905 (0.603, 1.358)	0.629
No Data Available	83%	0%	-	-	0%	-	-
**Maternal Diagnosis**	** **	** **	** **	** **	** **	** **	** **
Diabetes Mellitus in pregnancy	-	10%	2.045 (1.396, 2.995)	p<0.001	11%	1.605 (1.15, 2.24)	0.005
Diabetes Mellitus without complications	-	5%	2.093 (1.262, 3.47)	0.004	5%	1.935 (1.216, 3.08)	0.005
Hypertension in pregnancy	-	9%	1.745 (1.167, 2.61)	0.007	12%	1.377 (0.987, 1.92)	0.060
Complications at birth	-	43%	1.29 (0.988, 1.685)	0.061	45%	1.158 (0.923, 1.452)	0.204
OB-related perin trauma	-	41%	0.781 (0.592, 1.029)	0.078	39%	0.815 (0.645, 1.031)	0.088
Pelvic obstruction	-	2%	1.36 (0.552, 3.35)	0.503	2%	1.931 (1.02, 3.653)	0.043
**Infant Diagnosis**	** **	** **	** **	** **	** **	** **	** **
Nutritional diagnosis	-	0%	0 (0, 0)	0.083	0%	0 (0, 0)	0.000
Epilepsy/convulsions	-	1%	2.483 (1.053, 5.853)	0.766	1%	2.483 (1.053, 5.853)	0.038
Liver Diseases	-	10%	0.743 (0.492, 1.122)	0.153	10%	0.743 (0.492, 1.122)	0.158
Skin Diseases	-	11%	1.022 (0.735, 1.419)	0.252	14%	1.022 (0.735, 1.419)	0.899
Kidney Diseases	-	1%	1.144 (0.556, 2.356)	0.334	2%	1.144 (0.556, 2.356)	0.714
Circulatory Diseases	-	1%	2.386 (0.968, 5.88)	0.000	1%	2.386 (0.968, 5.88)	0.059

Additionally, we found that both BMI and weight for length z-score (at the last reading available and at the end of the second year) were strongly associated with obesity outcomes at age five. The characteristic tables for these features are summarized in Tables 4 and 5 for girls and boys, respectively. Our analysis validates previous findings that a number of variables during infancy have significant associations with obesity later in childhood, which falls in line with previous findings that weight early in life can predict weight later in life [33, 35, 37, 53].

**Table 4:** Individual feature associations for girls with obesity between ages 4.5 and 5.5.

Variable	Total Number	Total Average (SD)	% Obese (N)	Obese Average (SD)	% Not Obese (N)	Not Obese Average (SD)	p-value
Weight for Length Z-score (average 19 to 24 months)	1,347	1.042 (1.106)	22.7% (316)	1.899 (1.029)	77.3% (1,076)	0.79 (0.996)	p<0.001
BMI (average 19 to 24 months)	1,355	17.547 (1.786)	22.7% (318)	18.869 (1.818)	77.3% (1,083)	17.158 (1.578)	p<0.001
Weight for Length Z-score (latest available reading)	1,612	0.99 (1.166)	22.1% (368)	1.806 (1.135)	77.9% (1,297)	0.759 (1.066)	p<0.001
BMI (latest available reading)	1,624	17.509 (1.806)	22.1% (371)	18.734 (1.953)	77.9% (1,304)	17.161 (1.599)	p<0.001

**Table 5:** Individual feature associations for boys with obesity between ages 4.5 and 5.5.

Variable	Total Number	Total Average (SD)	% Obese (N)	Obese Average (SD)	% Not Obese (N)	Not Obese Average (SD)	p-value
Weight for Length Z-score (average 19 to 24 months)	1,392	1.042 (1.106)	23.5% (316)	1.899 (1.029)	79.9% (1,076)	0.79 (0.996)	p<0.001
BMI (average 19 to 24 months)	1,401	17.547 (1.786)	23.5% (318)	18.869 (1.818)	79.9% (1,083)	17.158 (1.578)	p<0.001
Weight for Length Z-score (latest available reading)	1,665	0.99 (1.166)	22.8% (368)	1.806 (1.135)	80.5% (1,297)	0.759 (1.066)	p<0.001
BMI (latest available reading)	1,675	17.509 (1.806)	22.8% (371)	18.734 (1.953)	80.3% (1,304)	17.161 (1.599)	p<0.001

### Obesity prediction using EHR and machine learning

For our binary obesity classification and regression models, we were able to achieve performance comparable to, or better than, similar cohort-based studies [32–36]. However, we are not able to compare our results to directly to Dugan et al. because of the differences in reporting methods. We found that our regression models outperformed their classification counterparts for predicting obesity at age five with data from the first two years of life. On average, AUC on the test set with a 95% confidence interval was 0.042 [0.031, 0.052] higher for girls, and 0.033 [0.023, 0.043] higher for boys in the regression task than the classification task. The difference is significant because the confidence intervals do not overlap. An overview of performance assessment can be seen in the Jupyter Notebook in S2 File.

The best performing model with the highest mean AUC for girls was LASSO regression on the full feature set with LASSO feature selection. LASSO looks for a sparse solution, therefore the model only utilized 35 features. Details of these features can be found in S1 Table. Similarly, the best performing multivariate model by highest mean AUC in the regression analysis for boys was LASSO using only EHR data without feature selection with only 144 features being utilized. A summary of these 144 features can be found in S2 Table. However, the performance of this model was consistently lower than the best single feature model, average WFL z-score between 19 and 24 months, whereas the other three single feature models performed comparably to the best multivariate model. The details of these analyses can be seen in the Jupyter Notebook in S3 File.

**Fig 2:**
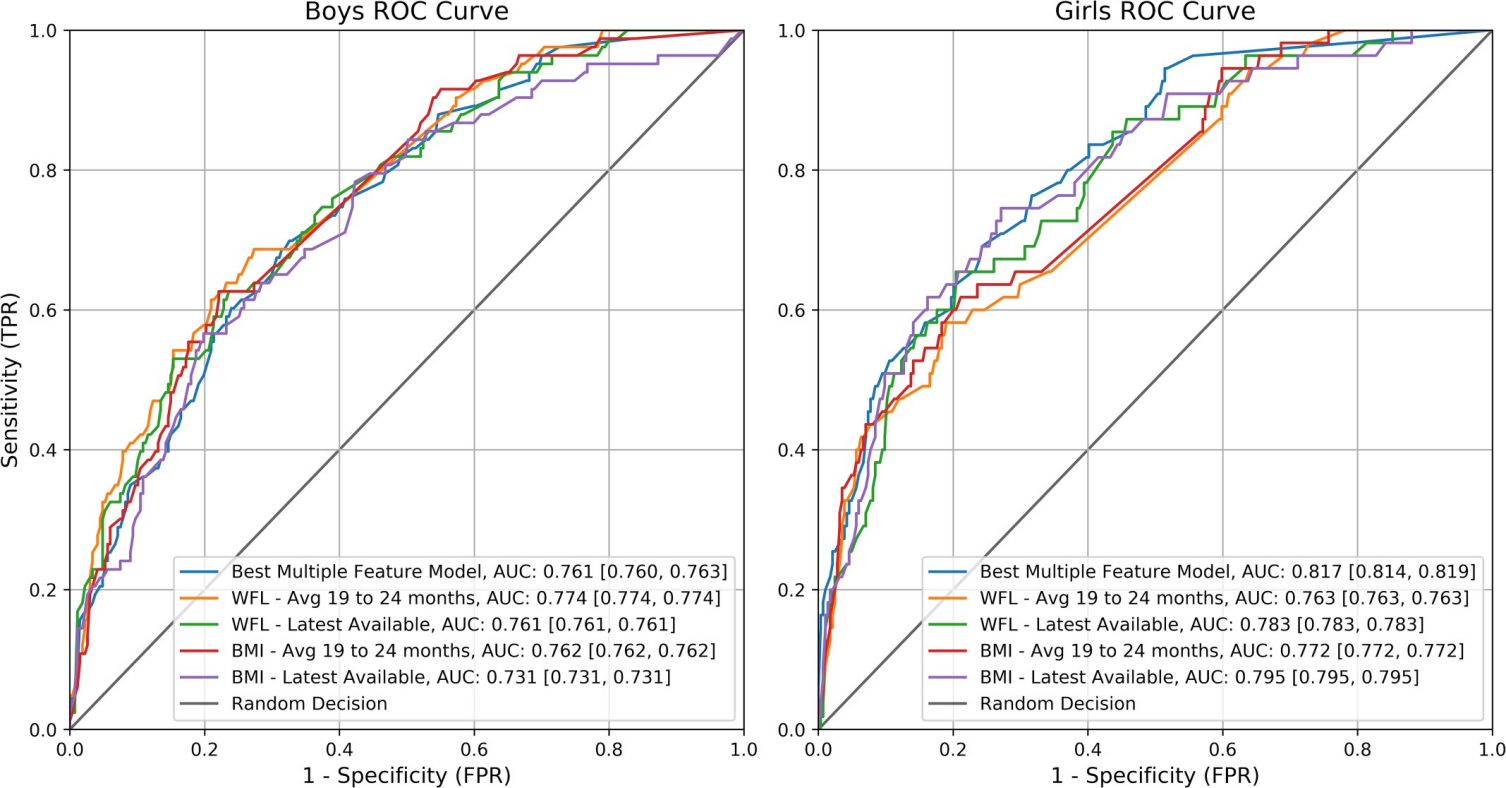
ROC curves for the top performing model compared to individual feature predictions.

**Fig 3:**
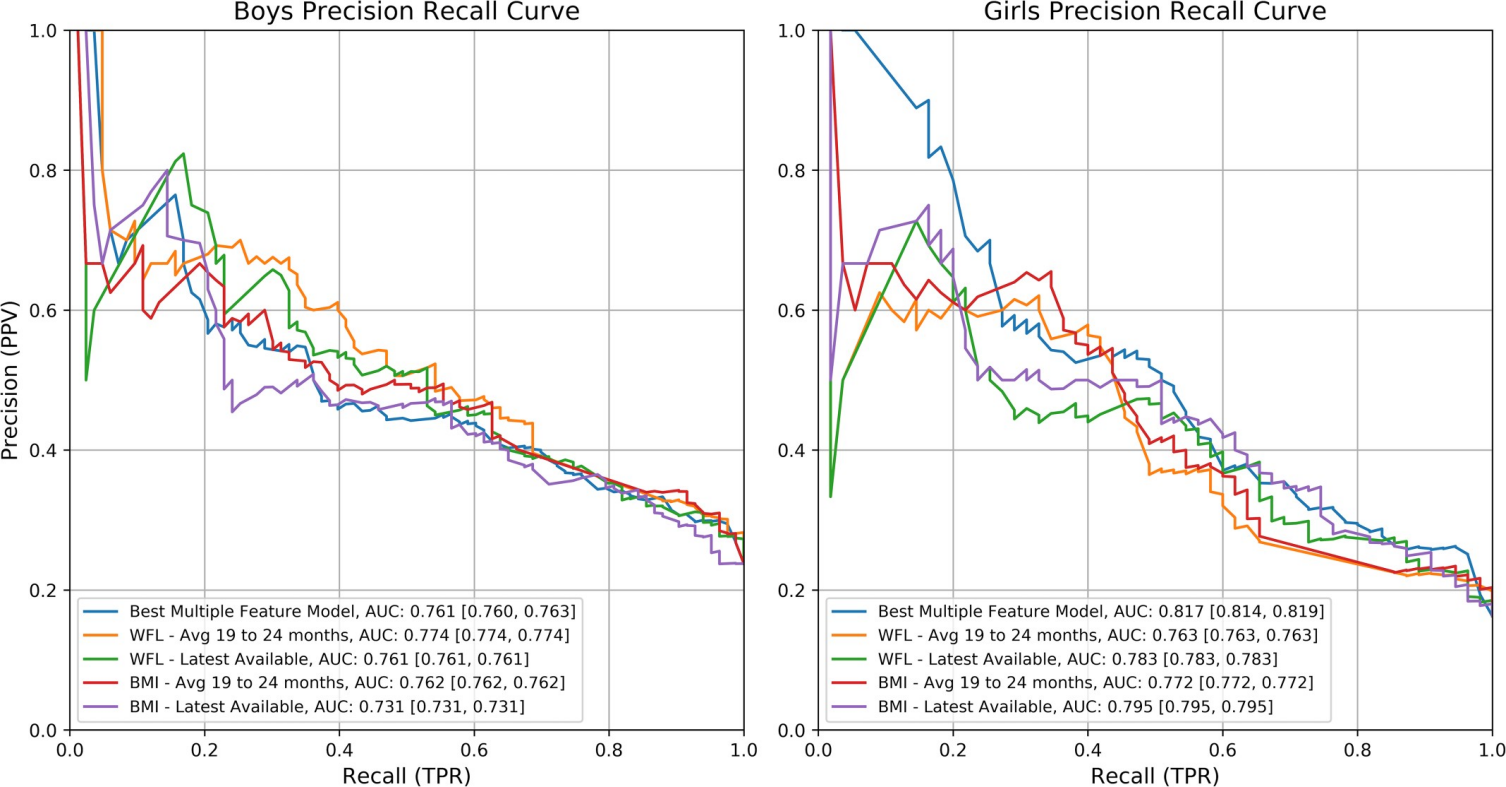
Precision recall curves for the top performing model compared to individual feature predictions.

Using our best performing multivariate model we were able to predict obesity on the test set with a mean AUC of 81.7% [81.4%, 81.9%] and 76.1% [76.0%, 76.3%] for girls and boys, respectively. Using these models we found that 34.3% and 28.1% of the variance of BMI at age five being explained for girls and boys respectively. The results for each of the models are shown in S3 Table and S4 Table for girls and boys, respectively. In Figs 2 and 3, we present the ROC curves and precision recall curves, respectively, for each of these highest performing models against the each of our individual feature models.

Threshold values for these plots can be seen in Table 6 and Table 7, for girls and boys respectively. We found that we had modest performance if the goal is to reach a high sensitivity, but when focusing on predicting children most at risk of having obesity at age five, then we are able to achieve higher levels of accuracy. While it is important to consider predicting obesity outright, we are focused on a mechanism for targeted intervention for high risk children, so we focus on the results where a high PPV is achieved. It can be seen that where PPV is high (at least 70%) our model accuracy as well as the Matthews Correlation Coefficient (MCC) are maximized. This means that both our accuracy and the tradeoffs between error types in our model are performing best for this task. This tradeoff is ideal when attempting to craft a more tailored intervention study where resources should be focused on children who are at a higher risk of developing obesity and not all children who may become obese.

**Table 6:** Performance tradeoffs for the best performing model for girls.

Sensitivity	PPV	Specificity	Accuracy	F1	MCC	N Obese (TP + FP)	N Not Obese (TN + FN)
0.145	0.889	0.996	0.858	0.250	0.352	9	330
0.200	0.786	0.989	0.861	0.319	0.126	14	325
0.291	0.571	0.958	0.850	0.386	0.030	28	311
0.418	0.535	0.930	0.847	0.469	0.021	43	296
0.491	0.519	0.912	0.844	0.505	0.018	52	287
0.600	0.371	0.803	0.770	0.458	0.007	89	250
0.691	0.355	0.757	0.746	0.469	0.006	107	232
0.800	0.293	0.627	0.655	0.429	0.004	150	189
0.891	0.261	0.511	0.572	0.403	0.003	188	151

**Table 7:** Performance tradeoffs for the best performing model for boys.

Sensitivity	PPV	Specificity	Accuracy	F1	MCC	N Obese (TP + FP)	N Not Obese (TN + FN)
0.084	0.700	0.989	0.774	0.151	0.071	10	340
0.205	0.567	0.951	0.774	0.301	0.021	30	320
0.301	0.543	0.921	0.774	0.388	0.015	46	304
0.398	0.458	0.854	0.746	0.426	0.008	72	278
0.506	0.442	0.801	0.731	0.472	0.007	95	255
0.602	0.435	0.757	0.720	0.505	0.006	115	235
0.699	0.397	0.670	0.677	0.507	0.005	146	204
0.795	0.346	0.532	0.594	0.482	0.003	191	159
0.904	0.306	0.363	0.491	0.457	0.003	245	105

The factor that emerged as most predictive for girls was the average maternal post-pregnancy weight despite having a weak AUC as its own predictor; however, weight and height related features for the infant were all but seven of the model’s 35. For the best performing multivariate model for boys, weight and BMI features made up 122 of the 144 total features. However, only 85 of the 144 features had beta coefficients greater than or equal to 0.001, with 71 of those features also relating to weight and BMI.

## Discussion

Since the Surgeon General's “Call to Action to Prevent and Decrease Overweight and Obesity" in 2001 [65], obesity and its causes has been the focus of numerous scientific studies [8, 66, 67]. Similarly, thousands of state-level policies have been enacted to encourage healthy lifestyles [68]. Despite the massive investments in money and effort so far, very few interventions have been effective at preventing obesity [69]. In this study, we used EHR and machine learning algorithms to identify young children with a high risk of developing obesity that could be specifically targeted for intervention. Using LASSO regression, we could predict obesity, between the ages of 4.5 and 5.5 years old on a held-out test set, achieving average AUC scores of 81.8% for girls and 76.1% for boys (Fig 1).

Some previous intervention studies have focused on known risk factors, such as maternal ethnicity [70, 71]. If we had used this broad cohort specification, such as that in Gross, et al. [18], as opposed to machine learning methods, our PPV would have been 18.3% for girls and 25.7% for boys (Jupyter Notebook in S2 File). This means that 81.7% of intervention targets, for girls, and 74.3% of the intervention targets, for boys, did not have much risk of becoming obese in the first place. Potentially, these broad inclusion criteria could be contributing to the small effects found in intervention studies, likely leading to the limited effectiveness of the interventions themselves. In contrast, with our full model, the achieved PPV (at 20% sensitivity) are 78% and 56% for girls and boys, respectively. This is significant because it allows for researchers to be able to set thresholds for inclusion in a study to measure the impact of an obesity intervention relative to the risk of developing obesity. High confidence predictions for future obesity (high PPV) capture less of the overall population that will develop obesity but those predictions will contain fewer false positives as opposed to predictions that lead to capturing a larger portion of the obesity developing population. The former approach would likely produce higher statistical power in a study because of the rebalanced distribution of false positives from previous studies, along with the added ability to measure effects relative to the risk level would allow for better understandings of where specific intervention methods are most effective.

We found significant differences in AUC performance between the best performing models and the most predictive factors for girls and boys. Other work has found similar differences [72] though it is not straightforward to determine the reason why this might be the case. These differences suggest boys and girls follow different growth trajectories and/or are subject to different obesity influencing factors, as can be seen in S1 Table and S2 Table. For instance, there was an environmental influence for predicting future obesity in girls, as can be seen with some census features existing in the selected features, as well as influence from maternal health variables. This suggests that there may be more external influences leading to childhood obesity in girls that can be tracked outside of growth measures. However, for boys, we found that nearly all of the selected features directly related to measures of obesity. Additionally, our study aligns with previous work that prior weight and obesity status can predict later in childhood obesity status [33, 35, 37, 53].

A limitation of our study is that our cohort is not demographically representative of NYC at large, coupled with a relatively small sample size. We expect that future studies incorporating bigger cohorts with more regionally representative demographics could further improve model performance. In addition, the size of our study sample through using a single health system was the likely the culprit for representation issues within the data set.

Another limitation, but also a feature of our study, was the noisy and incomplete nature of EHR datasets. Like most EHR data, we had many sparse records with low information content. For some features, such as newborn diagnoses, the rarity of a specified observation was inherent to the features themselves. For others, the sparsity of information within a feature came from not having complete patient history in the specific healthcare system. We underline that this is a feature of our approach, as we utilize the inherent redundancy of the EHR variables to become robust to certain level of data incompleteness.

A real-time, predictive health tracker, sitting on top of existing EHR systems (particularly those that were linked across systems), could be powered by models like ours, to alert clinicians of children at high risk of developing obesity with a goal of improving their decision-making process. To best achieve such a goal of real-time health tracking, denser datasets, summarizing a child and their mother’s entire medical history would enrich our feature space and potentially improve performance. The model presented here is a very promising step towards achieving this goal of using EHR for early identification of patients at-risk for developing childhood obesity.

In this study, we have shown that we are able to detect with reasonable accuracy which children will have obesity by age five with data from the first two years of life. While our available data, despite a large number of visits, is limited compared to traditional prospective studies with curated cohorts and expensive to collect data [33–35, 53, 73], our models perform just as well or better. We have been able to train accurate prediction models, demonstrating that real-life EHR data can be a useful tool in aiding childhood obesity intervention research, by allowing clinicians to select cohorts with higher future obesity prevalence, leading to more effective intervention studies and clinical trials, and, consequently, more targeted intervention programs and policies.

## Supporting information

S1 File Feature engineering data overview. This file provides an overview of the features used in the paper’s analyses. The file can also be viewed in the following link on our GitHub through Jupyter’s NBViewer: https://nbviewer.jupyter.org/github/NYUMedML/ObesityPY/blob/master/src/Pediatric_Obesity_Prediction_Feature_Data.ipynb.
(IPYNB)

S2 File Comparison of regression and classification models' notebook. This file demonstrates the methods and results used to compare the performance of regression and classification techniques for prediction. The file can also be viewed in the following link on our GitHub through Jupyter’s NBViewer: https://nbviewer.jupyter.org/github/NYUMedML/ObesityPY/blob/master/src/Pediatric_Obesity_Prediction_Regression_Classification_Comparison.ipynb.
(IPYNB)

S3 File Final regression analysis notebook. This file provides an overview of the final analyses performed. The file can also be viewed in the following link on our GitHub through Jupyter’s NBViewer: https://nbviewer.jupyter.org/github/NYUMedML/ObesityPY/blob/master/src/Pediatric_Obesity_Prediction_Regression_100_bootstraps.ipynb.
(IPYNB)

S1 Table Non-zero features for the top performing girl’s regression model, LASSO with the full feature set and LASSO feature selection. “*” indicates a feature whose unadjusted odds ratio is significantly greater than or less than 1.
(XLSX)

S2 Table Non-zero features for the top performing boys regression model, LASSO with all features excluding those at the community-level. “*” indicates a feature whose unadjusted odds ratio is significantly greater than or less than 1.
(XLSX)

S3 Table Regression AUC for all girls models tested. (XLSX)

S4 Table Regression AUC for all boys models tested. (XLSX)
