# Correction: Predicting childhood obesity using electronic health records and publicly available data

**DOI:** 10.1371/journal.pone.0223796

**Published:** 2019-10-07

**Authors:** Robert Hammond, Rodoniki Athanasiadou, Silvia Curado, Yindalon Aphinyanaphongs, Courtney Abrams, Mary Jo Messito, Rachel Gross, Michelle Katzow, Melanie Jay, Narges Razavian, Brian Elbel

The Funding statement is incorrect. The correct Funding statement is as follows: Rodoniki Athanasiadou was partially supported by RiskEcon® Lab for Decision Metrics @ Courant Institute of Mathematical Sciences NYU.
